# Minimally invasive direct coronary artery bypass grafting (MIDCABG) - our experience in Olomouc, Czech Republic

**DOI:** 10.1186/1749-8090-8-S1-P142

**Published:** 2013-09-11

**Authors:** P Santavy, V Lonsky, M Gwozdziewicz

**Affiliations:** 1 Dept. of Cardiac Surgery, Palacky University Teaching hospital, I.P.Pavlova 6, Olomouc, 779 00, Czech Republic

## Background

Minimally invasive direct coronary artery bypass grafting (MIDCAB) has become an interesting alternative to conventional surgery, especially in patients with isolated LAD stenosis unsuitable for PCI, in-stent restenosis or in all patients where avoidance of sternotomy and extracorporeal circulation can decrease the potential high risk of conventional CABG.

## Methods

To analyze the results of MIDCAB surgery at our department.

## Results

Between 2008-2013 (June) a total of 2316 CABGs were performed at our department, out of which 149 (6,4%) were MIDCABs. The mean age of pts was 67 years (±12), male/female ratio 89/60. Indications were as follows - isolated LAD stenosis 64, hybrid procedure (combination with PCI) 27, in-stent restenosis 4, palliative single vessel procedure 30, redo after previous cardiac surgery 9, different malignancy 8. Intraoperative conversion to full sternotomy 2 (1,3%), re-exploration for bleeding 2 (1,3%), 30-day mortality 2 (1,3 %).

## Conclusions

The MIDCAB is a safe and predictable surgical option for patients with single vessel coronary disease (LAD), who are not suitable for angioplasty. Other candidates who can benefit from miniinvasive procedure may be patients with multi vessel disease with the other coronaries occluded, selective redo surgeries, concomitant malignancies, severely reduced lung functions and overall reduced life expectancy. It can be performed with low mortality, frequency of conversion, re-exploration for bleeding and other postoperative complications.

